# Functional lateralization of arithmetic processing in the intraparietal sulcus is associated with handedness

**DOI:** 10.1038/s41598-020-58477-7

**Published:** 2020-02-04

**Authors:** Christina Artemenko, Maria A. Sitnikova, Mojtaba Soltanlou, Thomas Dresler, Hans-Christoph Nuerk

**Affiliations:** 1 Department of Psychology, University of Tuebingen, Tuebingen, Germany; 2 LEAD Graduate School & Research Network, University of Tuebingen, Tuebingen, Germany; 3 Department of Psychology, Pedagogical Institute, Belgorod National Research University, Belgorod, Russia; 4 Department of Psychiatry and Psychotherapy, University of Tuebingen, Tuebingen, Germany; 5 Research and Project Centre for Cognitive Neuroscience and Neurotechnologies, Belgorod National Research University, Belgorod, Russia

**Keywords:** Cognitive neuroscience, Learning and memory

## Abstract

Functional lateralization is established for various cognitive functions, but was hardly ever investigated for arithmetic processing. Most neurocognitive models assume a central role of the bilateral intraparietal sulcus (IPS) in arithmetic processing and there is some evidence for more pronounced left-hemispheric activation for symbolic arithmetic. However, evidence was mainly obtained by studies in right-handers. Therefore, we conducted a functional near-infrared spectroscopy (fNIRS) study, in which IPS activation of left-handed adults was compared to right-handed adults in a symbolic approximate calculation task. The results showed that left-handers had a stronger functional right-lateralization in the IPS than right-handers. This finding has important consequences, as the bilateral IPS activation pattern for arithmetic processing seems to be shaped by functional lateralization and thus differs between left- and right-handers. We propose three possible accounts for the observed functional lateralization of arithmetic processing.

## Introduction

One important indicator of functional lateralization in the brain is handedness. Handedness describes the preferential use of one of the hands with high accuracy and motor speed while performing skilled (i.e., culturally influenced activities such as handwriting) and unskilled actions based on spontaneous activities (e.g., picking up items)^1^. Both right- and left-handers represent the normal range of human diversity, but they differ in processing of various types of information and in the functional lateralization of the brain^2^.

The role of handedness as a behavioural reflection of functional lateralization was already investigated in neurocognitive domains such as language processing^3^ and spatial processing^4^. Language processing is left-lateralized in about 95% of right-handers, while left-handers either also show a left-lateralized (75%) or a right-lateralized or bilateral representation of language^5–7^. On the other hand, spatial processing is generally right-lateralized in right-handers and bilaterally or left-lateralized represented in left-handers^4,8^. Thus, functional lateralization is already established in other domains such as language and spatial processing, but was rarely studied in the domain of arithmetic processing so far.

### The Neural Representation of Arithmetic Processing

Brain activation associated with arithmetic processing relies on a fronto-parietal brain network^9^. Thereby, the core of number magnitude processing is considered to be the bilateral intraparietal sulcus (IPS) located in the parietal cortex between the superior parietal lobule (SPL) and the inferior parietal lobule (IPL) ^for dominant models and their extension see^^10–12^^; for important data see also^^13,14^. However, while the bilateral IPS is associated with arithmetic processing^15^, some studies reveal a more pronounced left hemispheric activation^16–21^. Brain stimulation studies confirmed the functional role of the bilateral IPS in arithmetic processing and more basic number processing^22–24^ with a larger involvement of the left IPS^22,25,26^ compared to the right IPS^27^.

This more pronounced left- than right-hemispheric IPS activation especially holds for symbolic arithmetic in Arabic notation compared to non-symbolic arithmetic^28^, which is supported by the basic differentiation of symbolic and non-symbolic number processing across modalities^29^. Different involvement of the left and right IPS were further found for approximate compared to exact calculation, while approximate calculation was found to be associated with stronger bilateral IPS activation than exact calculation^28,30,31^.

These findings lead to the question whether there might be a functional lateralization of arithmetic processing within the IPS. This means that the bilateral or predominantly left-lateralized IPS involvement in arithmetic processing might be true for right-handed individuals, who form about 90% of the human population^32^, but not for the human population as such. In particular, this might not hold for left-handed individuals, who are usually discarded from neuroscientific studies as nuisance factor and might show an opposite lateralization. Therefore, the current study addresses the issue, whether left-handers differ from right-handers in the lateralization of IPS activation during symbolic approximate calculation.

### The Role of Handedness in Arithmetic Processing

Behaviourally, there is no clear evidence for general performance differences between marked right-handers and left-handers in math^33^, although both right- and left-handers usually perform better than for example mixed-handers^34–36^. However, somewhat contrary to the above findings, professional mathematicians show on average a lower degree of handedness^37,38^. Besides, left- and right-handers were shown to differ in some basic numerical effects, as for example in the markedness effect^39,40^ and some spatial-numerical associations^41^^; but see^^39,40^, but not in the distance effect or compatibility effect^42^. In sum, there is no consistent empirical support for differences between right-handers and left-handers in math performance, because some findings also seem to depend on age, sex and task^34^.

Neurally, effects of handedness have not been systematically examined in the field of numerical cognition so far. Some neuroimaging studies, however, provide hints towards a difference in neural activation between left- and right-handers, although only very small samples of left-handers were analyzed (*n* = 8^16^, *n* = 7^43^, *n* = 3^44^). According to this weak evidence, in the prefrontal cortex right-handers show a stronger left-lateralization, while left-handers might show a bilateral or right-lateralized activation pattern^16,43,44^. In the parietal cortex, lateralization aspects are even less clear, because a left-lateralization for arithmetic processing might hold for both right- and left-handers^43^ or it might be just stronger in right-handers^44^. Moreover, lateralization seems to decrease with increasing arithmetic complexity^43^^; see also^^45^. Because of the essential role of the IPS in arithmetic processing, the relation of handedness to the functional lateralization of arithmetic processing needs to be resolved in this brain region.

### Objective

Functional lateralization of the brain was previously shown for other cognitive processes; however, it was not systematically investigated for arithmetic processing. Although arithmetic processing is considered to be bilaterally represented in the IPS, there are some indications of different functional roles of the two hemispheres. Therefore, the aim of the current study is to test functional lateralization of arithmetic processing in the IPS. As a well-established indicator of functional lateralization, we focus here on the relation between handedness and arithmetic processing, since handedness was found to be associated with some numerical effects^39,40^. Hence, we will investigate whether left-handers reveal a difference from right-handers in activation within the IPS during a symbolic approximate calculation task. As there are mostly no clear performance differences between left- and right-handers on the behavioural level, the current study addresses the issue whether functional lateralization can be detected on the neural level. In right-handers, symbolic arithmetic was found to be associated with activation of the left IPS and approximate calculation with bilateral IPS activation. The question of the current exploratory study is whether neuroimaging findings from right-handers can be generalized to human arithmetic processing in general or, as we expect, whether left-handers show less pronounced activation of the left IPS but instead more activation of the right IPS compared to right-handers because of differences in functional lateralization.

## Methods

### Participants

Seventy adults participated in the study, who were native German speakers with no history of neurological or mental disorders. Participants were excluded from analysis if they had an overall error rate exceeding 25% (*n* = 3) or because of missing neural data (*n* = 2) or noisy data (*n* = 10). Handedness was evaluated by the Edinburgh-Handedness Inventory, where the laterality index of handedness (LI_handedness_) was calculated according to the formula $(R-L)\div(R+L)\times 100$^46^. Individuals are considered as marked left-handers in the range of −100 to −40, as marked right-handers in the range of + 40 to + 100, and as mixed-handers in the range of −40 to +40. The resulting sample included 23 marked left-handers, 23 marked right-handers, and 9 mixed-handers (cf. Table 1). The handedness groups did not differ significantly in age, gender, or final math grade at school (cf. Table 1). Each participant gave written informed consent and received monetary compensation or student credits. The study was approved by the Ethics Committee of the Medical Faculty of the University of Tuebingen and conducted according to the ethical guidelines and principles of the international Declaration of Helsinki.

**Table 1:** Sample characteristics for left-, mixed- and right-handers.

	left-handers	mixed-handers	right-handers	statistics
LI_handedness_	−79.55 ± 29.53	−1.33 ± 24.61	76.34 ± 17.12	
age	24.05 ± 3.22	25.99 ± 4.10	24.12 ± 5.61	*F*(1, 52) = 0.82, *p* = 0.445
gender	15 f + 8 m	7 f + 2 m	19 f + 4 m	*F*(1, 52) = 0.93, *p* = 0.402
math grade	2.48 ± 0.90	2.78 ± 0.83	2.48 ± 1.47	*F*(1, 52) = 0.25, *p* = 0.782

*M*s and *SD*s are given for handedness in terms of LI_handedness_, for age in years, and for the final math grade at school (ranging from 1 [best] to 6 [worst]). *N*s of females (f) and males (m) are given for sex. Statistical analyses reflect ANOVAs with the between-subject factor handedness (left, mixed, right).

### Material

**Figure 1:**
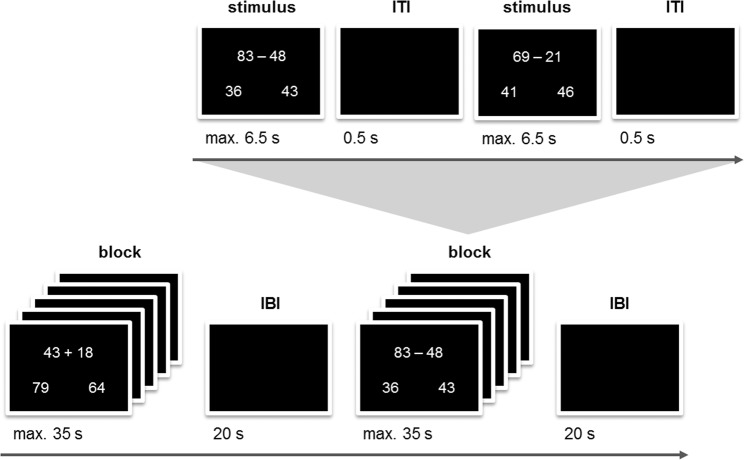
Approximate calculation task with a block design.

The approximate calculation task consisted of two-digit addition and subtraction problems. In a choice reaction paradigm each arithmetic problem was presented simultaneously with two solution probes^47^, whereby none of them represented the correct result of the arithmetic problem (cf. Fig. 1). This kind of task triggers approximate rather than exact calculation and relies on number magnitude processing^30,47^. The task was to choose the solution probe that was closer to the correct answer (target) by pressing the left or right Ctrl key on a standard computer keyboard with the left or right index finger, respectively. This procedure assured that motor activity should not differ between left- and right-handers. The target had a distance of ±1–3 to the correct result within the same decade and the distractor distance was either small (±4–8) or large (±14–18), whereby the direction of the distance from target and distractor to the correct result was the same.

For each combination of addition/subtraction with small/large distractor distance a stimulus set consisting of 25 arithmetic problems was created. Full decades (e.g., 30), ties within and between all numbers of an arithmetic problem (e.g., 55 + 16 or 25 + 45) were not included in the stimulus set. Addition problems with small and large distractor distance were matched for various stimulus properties^47^: the numerical size and parity of the operands, target, distractor and correct result; overall problem size; relative and absolute distances between target, distractor and correct result; need of a carry operation (or borrow operation in case of subtraction); decade crossing between target and distractor; the positions of the smaller operand and of the target. Subtraction problems were constructed as the inverse problems of addition (e.g., 46 + 38→84 − 38).

### Procedure

This study was part of a larger project; we focus here only on the data of the approximate calculation task. During the functional near-infrared spectroscopy (fNIRS) measurement, each participant was sitting in front of a computer in a dimly lit room. After fNIRS preparation and receiving instructions, the participant performed computerized tasks including the approximate calculation task during the fNIRS measurement.

In the approximate calculation task, all problems and solutions were presented in white on a black screen using Presentation software (Neurobehavioral Systems, Inc., Berkeley, CA, USA). The problems were embedded in a block design with a block length of 35 s and an inter-block interval of 20 s when the screen remained black (cf. Fig. 1). Blocks for each combination of addition/subtraction with small/large distractor distance were presented in randomized order in each of 5 runs (20 blocks in total). Each block started with 5 critical trials chosen from the respective stimulus set and was added up with additional filler trials chosen from an additional matched stimulus set. Trial order within each stimulus set was randomized for each participant. Each trial was terminated by button press or when the time limit of 6.5 s was reached. Termination was followed by an inter-trial interval of 0.5 s. Participants were encouraged to solve math problems as quickly and accurately as possible. Participants did not receive feedback as to the correctness of their response. Prior to the experimental trials, the participants solved 6 practice trials in order to become familiar with the task. The duration of the task was 20 min.

### fNIRS data acquisition

fNIRS recordings during the approximate calculation task were performed using the mobile near-infrared spectroscopy device NIRSport Model 88 (NIRx Medical Technologies, LLC, NY, the USA). Eight sources and eight detectors were mounted in an fNIRS cap according to the 10/20 system covering the parietal lobes of both hemispheres (9 channels per hemisphere) with centres in CP3 – P3 and CP4 – P4, respectively (cf. Figure S1 in the Supplementary Material). Two near-infrared laser beams with wavelengths of 760 and 850 nm were emitted. The sampling rate was 7.8125 Hz.

### Data analysis

All statistical analyses were conducted by using SPSS (IBM SPSS Statistics, version 25; IBM Corp., Chicago, IL, USA) and effect sizes were calculated according to Lakens^48^. For behavioural data analysis, only critical trials (and not filler trials) were entered into the analyses. Response time (RT) was regarded as the time interval from problem presentation on the screen to participants’ pressing one of two possible response keys. Only correct trials were included in RT analysis (exclusion of 11.56%), RTs beyond 3 *SD* of the participant’s *M* were repeatedly excluded (exclusion of 0.79%), and finally mean RT was calculated for each participant. The error rate (ER) was regarded as the proportion of incorrect or time-out responses to the total number of trials included in the analysis. RT and ER were compared between left-handers and right-handers by *t*-tests for independent samples. Note that the behavioural data of one participant could not be analyzed because of wrong button use (neural data of this participant was nevertheless included).

The relative concentration changes of oxygenated (O_2_Hb) and deoxygenated haemoglobin (HHb) were extracted from the fNIRS signal for each channel. fNIRS data pre-processing was performed by using custom MATLAB (The MathWorks, Inc., USA, version R2013a) scripts. Data pre-processing included interpolating noisy fNIRS channels by neighbouring channels (9.29%), excluding blocks with uncorrectable artefacts (3.09%), and bandpass filtering of 0.01-0.2 Hz. The signal was further corrected by correlation-based signal improvement (CBSI) according to the assumption that cortical activation is reflected by simultaneous increases in O_2_Hb and decreases in HHb^49^. Afterwards, the amplitudes of all blocks of 35 s were corrected to the baseline of 5 s before each block and averaged across all blocks resulting in the mean amplitude for each channel and participant.

As the region of interest (ROI), we focused only on the bilateral IPS being located between the SPL and IPL, i.e., channel L8 corresponding to the left IPS and channel R17 corresponding to the right IPS (for the location of the channels see Figure S1 and for the results of all channels see Figure S2 in the Supplementary Material). The positions of the channels are labelled by the corresponding brain region according to the automated anatomic labelling (AAL) atlas^50^ based on virtual-head-surface landmark measurements^51^. In the first analysis, a 2 × 2 ANOVA with the between-subject factor handedness (left vs. right) and the within-subject factor hemisphere (left vs. right) was conducted on the mean amplitudes.

In the second analysis, the laterality index of functional brain activation (LI_brain_), which should not be confused with the laterality index of the handedness questionnaire (LI_handedness_), was calculated for each participant according to the formula $(R-L)\div(abs(R)+abs(L))\times 100$, whereby *L* and *R* depict the mean amplitude within one channel (IPS) on the left (L8) and right (R17) hemisphere, respectively^52,53^. Thereby, negative values for LI_brain_ indicate a lateralization towards the left hemisphere and positive values indicate a functional lateralization towards the right hemisphere. LI_brain_ was compared between left-handers and right-handers by a *t*-test for independent samples. In the third analysis, in order to evaluate the relation between brain lateralization and the degree of handedness, LI_brain_ was correlated with LI_handedness_ in the whole sample, i.e., including left-, right- and mixed-handers. Note that while in the first two analyses only left- and right-handers were compared categorically, the third analysis, which used handedness as a continuous variable, could be conducted on all participants including mixed-handers.

## Results

### Behavioural results

Left-handers (*M = *3110 ms, *SD* = 614) and right-handers (*M = *2896 ms, *SD* = 629) did not differ significantly in RT [*t*(43) = 1.16, *p* = 0.253, *d* = 0.35]. Regarding ER, compared to left-handers (*M = *0.10, *SD* = 0.04), right-handers (*M = *0.13, *SD* = 0.06) made significantly more errors [*t*(36.72) = 2.41, *p = *0.021, *d* = 0.74]. Additionally, a correlation analysis was conducted to check whether this difference in ER was associated with lateralization in the brain. The results yielded no significant correlation between ER and LI_brain_ of left- and right-handers [*r*(43) = −0.15, *p* = 0.326], suggesting that the difference for LI_brain_ reported below does not seem to be influenced by the difference in performance between the groups.

### fNIRS results

**Figure 2:**
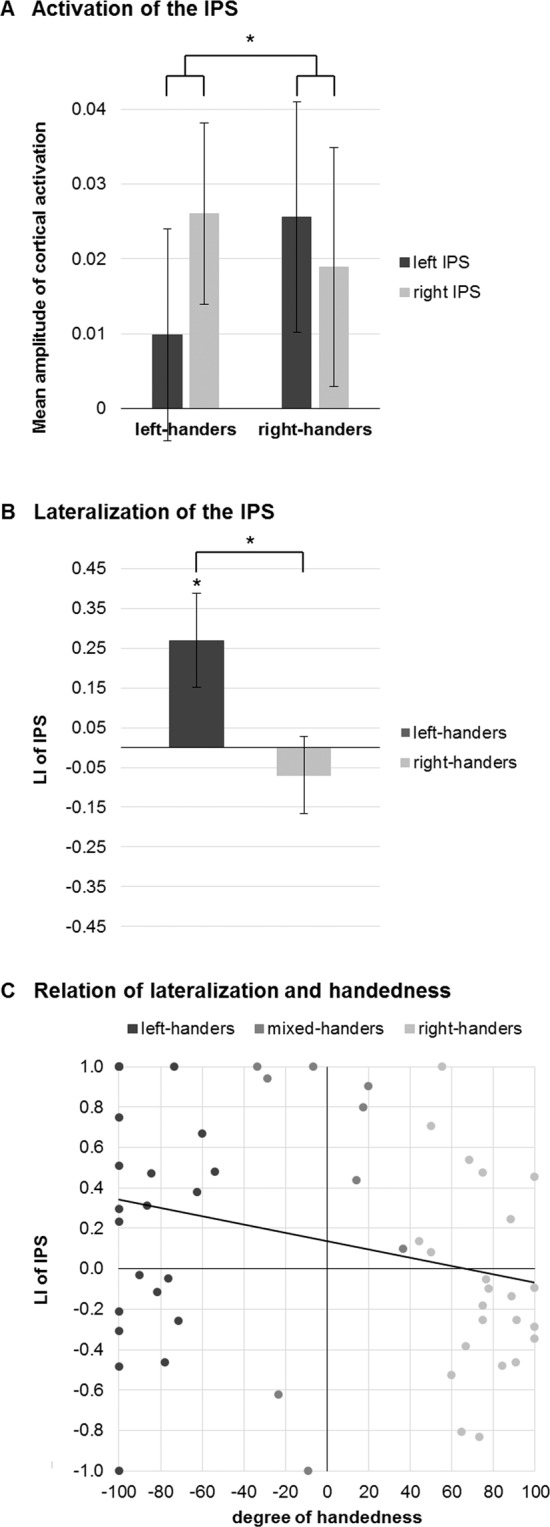
Relation of lateralization of the IPS and handedness during arithmetic processing. (**A**) Interaction of hemispheric activation of the IPS with handedness. (**B**) Lateralization of the IPS in left- and right-handers. (**C**) Correlation of lateralization in the IPS and degree of handedness. Negative values for LI_brain_ indicate left-lateralization and positive values for LI_brain_ indicate right-lateralization. Values for LI_handedness_ between −100 and −40 indicate left-handedness, between −40 and + 40 mixed-handedness and between + 40 and + 100 right-handedness. Significant effects are marked by stars (*). Error bars depict 1 *SE* of *M*.

The ANOVA for mean amplitudes revealed a significant interaction between handedness and hemisphere [*F*(1,44) = 4.06, *p* = 0.050, ${\eta }_{p}^{2}$ = 0.08], indicating that in left-handers activation was higher in the right IPS and in right-handers activation was higher in the left IPS (cf. Fig. 2A). The main effects of handedness [*F*(1,44) = 0.05, *p* = 0.830, ${\eta }_{p}^{2}$ < 0.01] and hemisphere [*F*(1,44) = 0.72, *p* = 0.401, ${\eta }_{p}^{2}$ = 0.02] were not significant.

The LI_brain_ for IPS activation differed significantly between left-handers and right-handers [*t*(44) = 2.21, *p* = 0.033, *d* = 0.67] (cf. Fig. 2B). This indicates that in comparison to right-handers [*t*(22) = −0.71, *p* = 0.485, *d* = 0.15], left-handers show a significant right-lateralization in the IPS [*t*(22) = 2.27, *p* = 0.033, *d* = 0.47], as revealed by post-hoc one-sample *t*-tests.

To further investigate the relation between handedness and lateralization in the IPS, a correlation analysis was conducted between LI_brain_ and the degree of handedness in terms of LI_handedness_ in the whole sample (including mixed-handers, because handedness was used as a continuous variable). A significant negative correlation was observed between LI_brain_ and LI_handedness_ [*r*(53) = −0.27, *p* = 0.044], indicating that a higher degree of left-handedness corresponds to a higher right-lateralization of IPS activation (cf. Fig. 2C), thus corroborating the results of the categorical analysis.

## Discussion

This study set out to test functional lateralization of arithmetic processing in the parietal cortex. In the domain of numerical cognition, weak evidence was found for functional lateralization of basic number processing^54^ and our finding adds to this by showing functional lateralization of arithmetic processing. In this way, the functional lateralization findings of these two studies seem to converge, even though different aspects of numerical processing (basic processing vs. arithmetic) were examined. This finding in the field of numerical cognition is not entirely surprising since functional lateralization was detected for other domains, such as motor function in terms of handedness, language processing^3^ and spatial processing^4^.

Most neurocognitive models of arithmetic processing assume a pivotal functional role of the bilateral IPS. However, virtually all these models and their supporting data have been based on studies in which predominantly right-handers were tested. Henceforth, the current study aimed at testing the functional lateralization of arithmetic processing by comparing the lateralization of the IPS during symbolic approximate calculation between left- and right-handers. In line with our hypothesis, we found a stronger right-lateralization in the IPS in left-handers than in right-handers. This supports the view that left-handers differ from right-handers in the lateralization of arithmetic processing in the IPS, so that there is an association between handedness and the neural representation of arithmetic. This result suggests that previous findings derived from right-handers cannot be readily generalized to the human population as such, and, in particular, not to left-handers. Consequently, existing models of arithmetic processing need to be extended to account for functional lateralization associated with handedness. Our data seem to indicate that the bilateral IPS activation for arithmetic processing seems to be shaped by functional lateralization: the importance and the roles of the left and the right IPS for arithmetic seem to be different in right-handers and left-handers.

Thereby, we need to acknowledge that the power of the main finding in our study was not so high (power of 0.60). Nevertheless, when integrating the weak evidence of functional lateralization of basic number processing in general^54^ together with the current finding of functional lateralization of arithmetic processing, there is cumulative evidence for the relation of handedness and lateralization in the IPS in the domain of numerical cognition, which would be a very important finding for neurocognitive models of numerical processing and beyond. However, we also wish to make clear that future research on lateralization in numerical cognition needs to substantiate these two findings by using better powered study designs, because the observed effects were not large.

The crucial question, however, is why arithmetic processing might show such a functional lateralization within the IPS. We offer different accounts of this issue, which might guide future research on the understudied topic of functional lateralization in numerical and arithmetic neurocognition.

### The embodiment account of functional lateralization in number processing

In the last years, a growing body of literature has suggested that even basic numerical cognition is embodied^55–57^^, for embodied trainings see^^58^. In particular, the use of hands and fingers has been postulated to influence numerical cognition, such as spatial-numerical associations^41,59–61^, magnitude comparison^55^, or also mental arithmetic^62^. Importantly, embodied cognition has even been suggested to influence the neural representation of numbers and operations on numbers^63,64^. For example, counting, as one of the most basic numerical skills, is associated with the excitability of motor circuits for hands^65^.

Against this background, it is conceivable that functional lateralization of arithmetic processing might be explained by the influence of the dominant hand used during the acquisition of symbolic arithmetic, which might lead to a contralateral hemispheric dominance to the preferentially used hand see also^41^. The underlying mechanism might be a co-lateralization of the motor activities, such as handwriting or finger counting, preferably conducted with the dominant hand and therefore be related to the developing cognitive skills in terms of symbolic arithmetic^66^. If the basic numerical representations on a neural level are indeed influenced by the preferentially used hand (as suggested for finger counting^64^), then a differential functional lateralization for left-handers and right-handers is not surprising. In particular, this account would explain the relatively stronger activation of the right IPS in left-handers (compared to right-handers), because the right hemisphere is contralateral to the dominant hand in left-handers.

Supporting the embodiment account of functional lateralization, the degree of handedness was correlated with functional lateralization of the IPS for arithmetic processing. Going beyond the group differentiation of left- and right-handers, the degree of handedness reflects an important factor that can influence cognitive abilities^3,8^. Here, with increasing degree of left-handedness, a stronger right-lateralization of the IPS was observed, which is in line with the embodiment account of functional lateralization.

### Co-lateralization of different neurocognitive functions: Associations of number with language and space

On the one hand, numbers are closely related to different dimensions of space like their spatial direction or spatial extension^67–69^, for a review and special issue on the topic see^70^. For instance, larger numbers are associated with the right side of space and smaller numbers with the left side of space in Western societies^71^. Since human infants^72^ and even newborns^73^ as well as other species^74,75^ were found to express space-number associations, the close relation between numbers and space might be even innate.

On the other hand, numbers are also related to different types of linguistic attributes ^for a review and special issue on this topic see^^76^. For instance, the grammatical language structure in number words seems to determine how easy or difficult it is to acquire numbers early in development^77^. Even in adulthood, language attributes like reading direction^42,78^ or the lexical composition of number words influence numerical cognition^76,79^. In sum, number processing has multiple relations to spatial and language processing on a behavioural level and can be traced back early in development.

In general, a co-lateralization of spatial and language processing has been observed. For instance, language processing and spatial attention were shown to be controlled by opposite hemispheres^80^. While language is considered to be left-lateralized in about 95% of right-handers and 75% of left-handers, the rest 25% of left-handers demonstrate either a right-lateralized or bilateral representation of language^5–7^. Although spatial processing is generally considered to be lateralized to the right hemisphere in right-handers^4^, it was shown that left-handers with right hemispheric dominance for language had left-hemispheric dominance for spatial attention^80^. If numerical cognition builds on spatial processing^81^ or linguistic processing^77^, the functional lateralization of these basic cognitive functions may lead to a similar co-lateralization of number processing.

The co-lateralization of different cognitive functions requires further investigation in the future in order to determine the position of arithmetic processing in relation to the processing of language and space. The current study is limited in this regard, since language dominance was not assessed and the observed difference in handedness might reflect language lateralization to a certain extent^82^. However, if the co-lateralization account also holds for arithmetic processing, a lateralization of arithmetic seems reasonable because of the interrelation of arithmetic with language and spatial processing.

### Endpoint of different developmental trajectories

**Figure 3:**
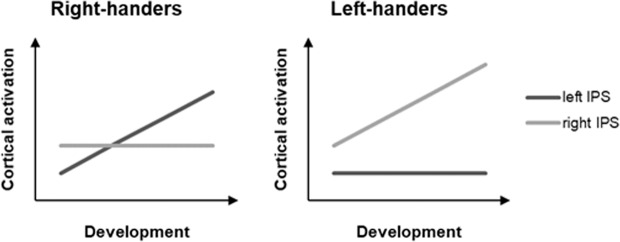
Possible functional lateralization of the IPS for arithmetic processing during development. Based on the literature, activation of the left IPS increases and activation of the right IPS is stable during development in right-handers. Contrary, it is assumed here that left-handers show the opposite pattern resulting in right-lateralization of the IPS for arithmetic processing.

The different lateralization of arithmetic processing in right-handers and left-handers might be further supported by developmental findings for the IPS. Namely, there might be different trajectories of development of the two hemispheres in left- and right-handers. In right-handers, activation in the left IPS for arithmetic processing increases during development^83–86^^, but see^^44,87^. On the other hand, there is much less evidence for a similar activation increase in the right IPS^83,86^ and it might even be that there is no such activation change in the right IPS during development^88,89^. Since no literature on the development of the left and right IPS exists for left-handers, we can only speculate and hypothesize the opposite pattern for left-handers. This means an activation increase in the right IPS during arithmetic development for left-handers (cf. Fig. 3), explaining our finding of a right-lateralization in left-handers compared to a left-lateralization in right-handers for arithmetic processing. In sum, the functional lateralization of arithmetic processing might be a result of increased activation in the left IPS in right-handers and increased activation in the right IPS in left-handers during arithmetic development (cf. Fig. 3).

It is important to note that all three accounts are not mutually exclusive. For instance, this proposed developmental account of functional lateralization for arithmetic processing is supported by both the embodiment account as well as the co-lateralization account. In regards to embodied cognition, due to the strong relation between motor activities (like handwriting for symbolic arithmetic or even number-related motor activities like finger counting) with the right hand and contralateral left-hemispheric activation, arithmetic processing might be specialized more to the left parietal cortex in right-handers during development, while in left-handers arithmetic processing might be specialized to the right parietal cortex. In regards to co-lateralization, number processing was found to be initially represented in the right IPS^90–92^ and magnitude relates more to its visuo-spatial representation, which was shown to be right-lateralized as well. During development, arithmetic processing becomes more related to verbal representations and thus left-lateralization for right-handers and right-lateralization for left-handers becomes more prominent because of the hemispheric dominance for language processing. The proposed developmental model (cf. Fig. 3) reflects a proposal for the development of functional lateralization of arithmetic processing, which needs to be empirically evaluated in future research.

For right-handers, we did not find evidence for a strong left-lateralization within the parietal cortex. This might be due to the point that approximate calculation in comparison to exact calculation was shown to be rather bilaterally represented in the parietal cortex in right-handers^30,31^. Furthermore, the complexity of the arithmetic problems used in the current study was relatively high, so it could lead to less pronounced left-lateralization^43,45^. However, these explanations would also hold for left-handers and thus contradict the detected lateralization effect in left-handers. Therefore, the explanation might be derived from the hypothetical model (cf. Fig. 3), where the degree of lateralization in the IPS is expected to be larger in left-handers than in right-handers (given a similar slope of IPS development in right-handers and left-handers).

### Limitations

In the current study, we did not control for saccades during calculation so that a possible different pattern of saccades in left- and right-handers might have had an impact on the lateralization of neural activation. On the one hand, it represents a general limitation for neuroimaging research that saccades might differ between groups or conditions (although the same stimuli were shown to both groups). On the other hand, this might represent a specific problem for research on arithmetic because of operational momentum effects^93–95^ (although the stimulus material consisted of both addition and subtraction problems in equal parts, so that this very effect is not a problem for our study). However, we cannot exclude that other sources of eye-movement behavior differ for left- and right-handers, although we do not find strong indication in the literature that this should be the case in our paradigm. Nevertheless, a difference in saccades might be an alternative explanation for the lateralization effects observed in our study, which needs future investigations.

### Conclusions

Functional lateralization, as indicated by handedness, – as previously shown for other domains like language and space – was demonstrated here for arithmetic processing. Our results refine the view that the bilateral IPS involvement for arithmetic processing, usually observed in empirical studies and postulated by the dominant models of numerical cognition, is similar for all humans. Namely, we observed left-handers to have a stronger right-lateralization in the IPS for symbolic approximate calculation compared to right-handers. We proposed three different accounts for this functional lateralization:

(i) The embodiment account: Because of the embodied influences even on basic numerical representations, the preferred use of the dominant hand in number-unrelated, but also number-related activities (finger counting) might determine the functional lateralization of arithmetic processing.
(ii) The co-lateralization account: Because numbers are tightly linked to spatial and language processing, the functional lateralization of these cognitive functions may be associated with the functional lateralization of arithmetic processing.
(iii) The developmental account: Because of individual differences in developmental trajectories, which determine lateralization in other behaviours such as handedness, language, and spatial processes, the functional lateralization of arithmetic processing at the endpoint of this development may also be different.

Note that these three accounts are not mutually exclusive. We proposed how they could function and also how they could be tested in future. We believe that this endeavour is of utter importance for several reasons. First, the phenomenon of left-handedness cannot be neglected in research and models of arithmetic processing. Second, functional lateralization is considered to be beneficial for cognitive skills^8^ and thus understanding the mechanisms might be beneficial for arithmetic education. Finally, such research addresses general mechanisms of neurocognitive functioning (embodiment, co-activation, development), for which functional lateralization research provides a critical test, especially if it holds even for abstract representations such as number magnitude in the IPS. Since the power and design of our study were limited, we recommend more powerful studies investigating several aspects of arithmetic processing (e.g., symbolic vs. non-symbolic, exact vs. approximate calculation) and functional lateralization (e.g., handedness, language dominance) to follow-up. In any case, we believe that this study on functional lateralization of arithmetic processing provides a good starting point for a future research line.

## Supplementary information

Supplementary Material.

## Data Availability

The datasets generated during the current study are not publicly available due to ethical restrictions but are available from the corresponding author on reasonable request.
