# Supplementary material for: Functional lateralization of arithmetic processing in the intraparietal sulcus is associated with handedness
Source: Sci Rep. 2020 Feb 4;10:1775. doi: 10.1038/s41598-020-58477-7 (PMC7000739; doi:10.1038/s41598-020-58477-7)
Supplement: Supplementary file 1 — Supplementary Material. [file 41598_2020_58477_MOESM1_ESM.pdf]

SUPPLEMENTARY MATERIAL

Paper

Functional lateralization of arithmetic processing in the intraparietal sulcus is associated with handedness

Authors

Christina Artemenko, Maria A. Sitnikova, Mojtaba Soltanlou, Thomas Dresler, Hans-Christoph Nuerk

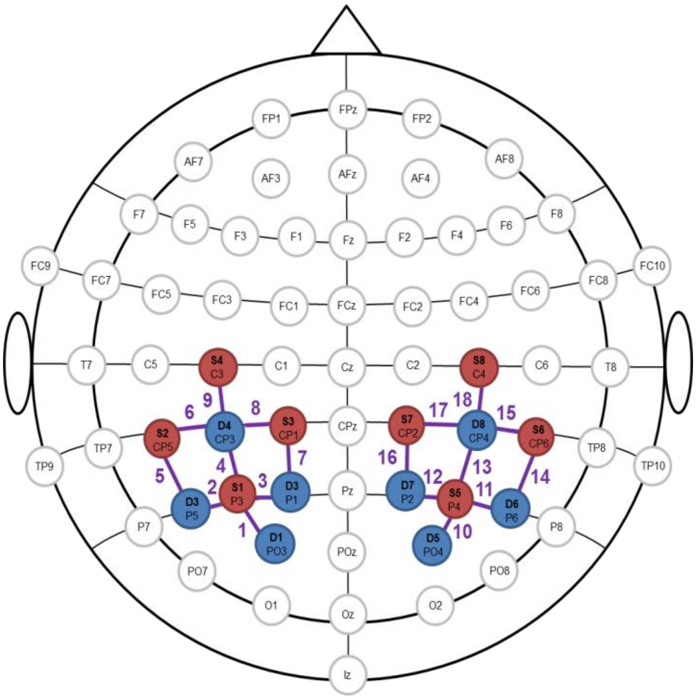

Figure S1. fNIRS probeset over the left and right parietal cortex. Positions of the optodes (red: sources; blue: detectors) are indicated according to the international 10-20 system and channels are labeled by numbers.

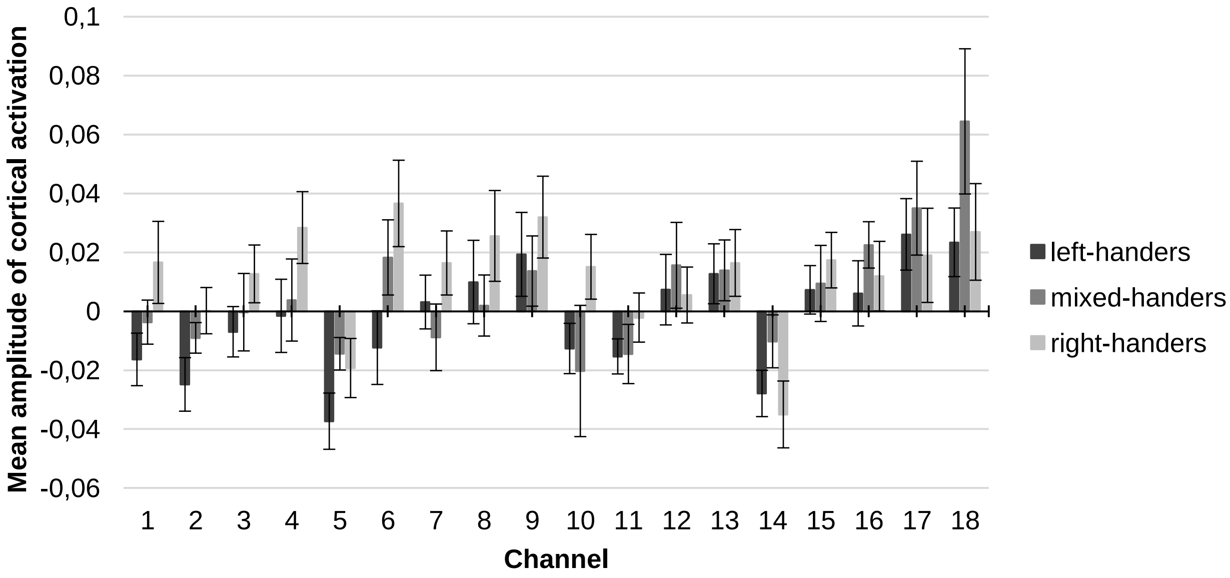

Figure S2. Average activation for each channel in left-, mixed- and right-handers. Error bars depict 1 *SE* of *M*.
